# Joint Formal Comment for PLGE and Commentary for G3

**DOI:** 10.1534/g3.120.401759

**Published:** 2020-10-27

Two recent papers by Gustavo Aguirre’s and Hannes Lohi’s groups have been published describing the genetics of Progressive Retinal Atrophy (PRA) in Miniature Schnauzers. This recessively inherited condition is a serious problem in companion animals, and also is a model for human retinitis pigmentosa. The first paper by Murgiano *et al.* 2019 (G3) proposed a complex structural variant affecting the coding region of the *PPT1* gene as causal, but some dogs were apparently homozygous for the variant and did not exhibit disease, suggesting incomplete penetrance. A more recent study by Kaukonen *et al.* (PLoS Genet 2020) identified the same locus, but identified a different candidate causal variant—a SNV in an intron of the *HIVEP3* gene, which lies ∼1.5 Mbp away from *PPT1*. In the Kaukonen *et al.* study, the candidate causal variant appeared completely penetrant, although the Murgiano *et al.* study reported 5 cases that were not homozygous for the *HIVEP3* variant, but were homozygous for the *PPT1* risk haplotype.

The different conclusions drawn in the two published papers have facilitated discussions and comparisons of the datasets between the groups, and some re-analyses have been conducted to test the presented alternate hypotheses of the causal candidate genes and variants. Importantly, these re-analyses reveal new insights which affect the interpretation of the data and related conclusions. We take this opportunity to summarize our analyses in a joint statement.

Key observations affecting the interpretations and conclusions in both studies relate to: (1) discovery of the non-mutated risk haplotype as described by Kaukonen et al.; and (2) the realization that is challenging to determine the zygosity of the complex *PPT1* structural variant without high quality WGS data.

In the work from Kaukonen *et al.*, a heterozygous obligate carrier dog was found to be homozygous for SNV markers previously described by Murgiano *et al.* as associated with the condition, revealing that the PRA mutation is recent, with both mutant and non-mutant chromosomes with the same haplotype segregating in Miniature Schnauzers. This same obligate carrier was originally inferred to be homozygous for the *PPT1* structural variant based on SNV genotyping; however, a new coverage-based analysis of WGS data revealed that the dog is actually heterozygous for the *PPT1* structural variant (and is also heterozygous for the *HIVEP3* variant).

Thus, the *genetic* evidence is unable to distinguish between the *HIVEP3* and *PPT1* variants as potential causes of PRA. Furthermore, the SNV markers initially proposed in Murgiano *et al.* are not reliable to determine the zygosity of the complex *PPT1* structural variant, or to reach conclusions regarding its potential penetrance. Although functional considerations favor causality of the coding *PPT1* structural variant over the intronic *HIVEP3* SNV, an efficient and inexpensive means of genotyping the *PPT1* structural variant has not been developed, limiting our ability to reach final conclusions about the true causal gene. It is also possible but unlikely that PRA is caused by a different variant in the mapped interval that could not be identified by whole genome sequencing.

For the purposes of diagnosis by breeders and veterinarians, the *HIVEP3* variant may be used in a genetic testing environment until final conclusions of the causal variant are made and a robust method to genotype the *PPT1* structural variant becomes available. Importantly, when testing the *HIVEP3* variant one should bear in mind that if *HIVEP3* is not the causal gene, recombination between *HIVEP3* and the causal variant would produce incorrect test results.

Sincerely yours,

**Table:** 

Leonardo Murgiano*	Maria Kaukonen
Doreen Becker	Ileana B. Quintero
Dina Torjman	Abdul K. Mukarram
Jessica K. Niggel	Marjo K. Hytönen
Ausra Milano	Saila Holopainen
Cheryl Cullen	Kaisa Wickström
Rui Feng	Kaisa Kyöstilä
Fan Wang	Meharji Arumilli
Vidhya Jagannathan	Sari Jalomäki
Susan Pearce-Kelling	Carsten O. Daub
Martin L. Katz	Juha Kere
Tosso Leeb	Hannes Lohi*
Gustavo D. Aguirre*	the DoGA Consortium
*corresponding authors	

